# Clinical and Biochemical Features in a Patient With *Mitochondrial Fission Factor* Gene Alteration

**DOI:** 10.3389/fgene.2018.00625

**Published:** 2018-12-07

**Authors:** Alessia Nasca, Francesca Nardecchia, Anna Commone, Michela Semeraro, Andrea Legati, Barbara Garavaglia, Daniele Ghezzi, Vincenzo Leuzzi

**Affiliations:** ^1^Unit of Medical Genetics and Neurogenetics, Fondazione IRCCS Istituto Neurologico Carlo Besta, Milan, Italy; ^2^Unit of Child Neurology and Psychiatry, Department of Human Neuroscience, Sapienza University of Rome, Rome, Italy; ^3^Division of Metabolism and Research Unit of Metabolic Biochemistry, IRCCS, Bambino Gesù Children's Hospital, Rome, Italy; ^4^Department of Pathophysiology and Transplantation, University of Milan, Milan, Italy

**Keywords:** mitochondrial fission factor, MFF, epileptic encephalopathy, leigh syndrome, mitochondrial disorders, mitochondria, peroxisome

## Abstract

Mitochondrial Fission Factor (MFF) is part of a protein complex that promotes mitochondria and peroxisome fission. Hitherto, only 5 patients have been reported harboring mutations in *MFF*, all of them with the clinical features of a very early onset Leigh-like encephalopathy. We report on an 11-year-old boy with epileptic encephalopathy. He presented with neurological regression, epileptic myoclonic seizures, severe intellectual disability, microcephaly, tetraparesis, optic atrophy, and ophthalmoplegia. Brain MRI pattern was compatible with Leigh syndrome. NGS-based analysis of a gene panel for mitochondrial disorders revealed a homozygous c.892C>T (p. Arg298^*^) in the *MFF* gene. Fluorescence staining detected abnormal morphology of mitochondria and peroxisomes in fibroblasts from the patient; a strong reduction in MFF protein levels and the presence of truncated forms were observed. No biochemical alterations denoting peroxisomal disorders were found. As reported in other disorders affecting the dynamics of intracellular organelles, our patient showed clinical features suggesting both mitochondrial and peroxisomal impairment. High levels of lactate in our case suggested an involvement of the energetic metabolism but without clear respiratory chain deficiency, while biomarkers of peroxisomal dysfunction were normal. We confirm that *MFF* mutations are associated with epileptic encephalopathy with Leigh-like MRI pattern.

## Introduction

Mitochondria are dynamic organelles and their morphology results from a finely tuned balance between fusion and fission events. These processes are particularly important for the maintenance of the cellular homeostasis: fission in particular allows quality control over the mitochondrial network, by separating defective mitochondria that are then targeted to mitophagy, and it is mediated by a series of nuclear-encoded proteins. Impairment in mitodynamics has been associated with diverse pathological conditions, including monogenetic forms of mitochondrial disorders caused by mutations affecting key players of this system. Dynamin-related protein 1 (DRP1, encoded by the *DNM1L* gene–MIM603850) is the main effector of mitochondrial fission but it also contributes to the division of peroxisomes (Li and Gould, 2003). DRP1 is mainly distributed in the cytoplasm and need proper cellular signals to be recruited to the mitochondria where it multimerizes creating a ring-like structure that separates the organelle. Several DRP1 receptors and recruitment factors on the outer mitochondrial membrane have been identified, including MFF (Mitochondrial fission factor–MIM614785), FIS1/TTC11 (Mitochondrial fission 1–MIM609003), GDAP1 (ganglioside-induced differentiation-associated protein 1–MIM606598) and MIEFs (Mitochondrial Elongation Factors; i.e., MIEF1-MIM615497 and MIEF2-MIM615498, also known as MiD51/MiD49). Notably, mutations in genes encoding some of these proteins have been associated with human disorders. For instance, genetic defects in *DNM1L* result in phenotypes varying from autosomal dominant optic atrophy to severe early onset mitochondrial encephalopathy, while mutations in *GDAP1* have been linked to forms of Charcot Marie Tooth Disease (Archer, 2013).

The outer membrane protein MFF is an essential component of the conserved machinery for membrane fission of mitochondria and peroxisomes, and presumably the major DRP1 receptor for organelle fission. Knockdown of *MFF* results in mitochondrial elongation and formation of tubular peroxisomes (Otera et al., 2010; Itoyama et al., 2013). Immunoprecipitation studies suggested MFF is part of a high-molecular weight complex including other proteins but not DRP1 (Gandre-Babbe and van der Bliek, 2008).

Biallelic mutations in *MFF* have been reported to be the cause of “Encephalopathy due to defective mitochondrial and peroxisomal fission 2” (EMPF2; MIM#617086), an extremely rare, autosomal recessive disorder characterized by delayed psychomotor development, severe hypotonia, and abnormal signals in the basal ganglia (Shamseldin et al., 2012; Koch et al., 2016).

We report here on the clinical and biochemical features of an 11-year-old Italian boy with epileptic encephalopathy, in whom we found a homozygous nonsense mutation in the *MFF* gene.

## Methods

### Genetic Studies

Informed consent for genetic analyses was obtained from the child's parents. The study was approved by the Ethical Committee of the Neurological Institute Besta. Written informed consent was obtained from the guardian of the patient for the publication of this case report. Genomic DNA was extracted from peripheral blood using standard method. Next generation sequencing of a panel of 230 nuclear genes associated with mitochondrial diseases was performed by MiSeq (Illumina), followed by bioinformatics analysis, as previously described (Legati et al., 2016).

### Immunoblotting

For western blot analysis, fibroblasts from patient and controls were pelleted and solubilized in RIPA buffer with protease inhibitors; 40 μg of protein was loaded for each sample in 12% denaturing sodium-dodecyl sulfate polyacrylamide gel electrophoresis (SDS-PAGE). Polyclonal antibodies against DRP1 (D6C7, #8570, Cell Signaling, 1:1000) and against MFF (17090-1-AP, Proteintech, 1:1000), and a monoclonal antibody against GAPDH (#MAB374, Millipore, 1:1000) were used.

### Fluorescence Microscopy

Skin fibroblasts were cultured in a 37°C incubator with 5% CO_2_, in either 25 mM glucose or 5 mM galactose Dulbecco's Modified Eagle Medium-DMEM (Euroclone) supplemented with 10% FBS, 1% L-glutamine and 0.2% sodium pyruvate. For visualization of the mitochondrial network, the mitochondrial fluorescent dye MitoTracker Red-CMXRos (Invitrogen) was added to culture media at a final concentration of 50 nM for 30 min and then images were acquired, in live cells, with a confocal microscope (Leica TSC-SP8). For visualization of peroxisomes, after fixation and permeabilization, cells were incubated with a polyclonal antibody anti-PMP70 (Millipore, 1:200), followed by a fluorescently labeled secondary antibody Alexa Fluor 488 (Invitrogen, 1:1000); then images were acquired with a confocal microscope (Leica TSC-SP8) using a 63x oil objective.

Analysis of mitochondrial network and peroxisomal morphology was conducted using the image processing package ImageJ (Fiji). Images were then binarized, thresholded, and subjected to particle analysis to acquire form factor (“circularity”: 4π^*^area/perimeter^2^). A value of 1.0 indicates a perfect circle; values approaching to 0.0 indicate increasingly elongated shapes. Data were analyzed using unpaired two-tailed Student's *t*-tests.

## Results

### Case Study

The proband was an 11 year-old Italian boy born from consanguineous (first cousins) parents after a pregnancy characterized by intrauterine growth retardation. The pedigree included an older brother with an early onset severe epileptic encephalopathy, who died at 16 years, and another healthy brother. Moreover, a previous pregnancy of the mother resulted in a spontaneous miscarriage in the first trimester.

**Figure 1:**
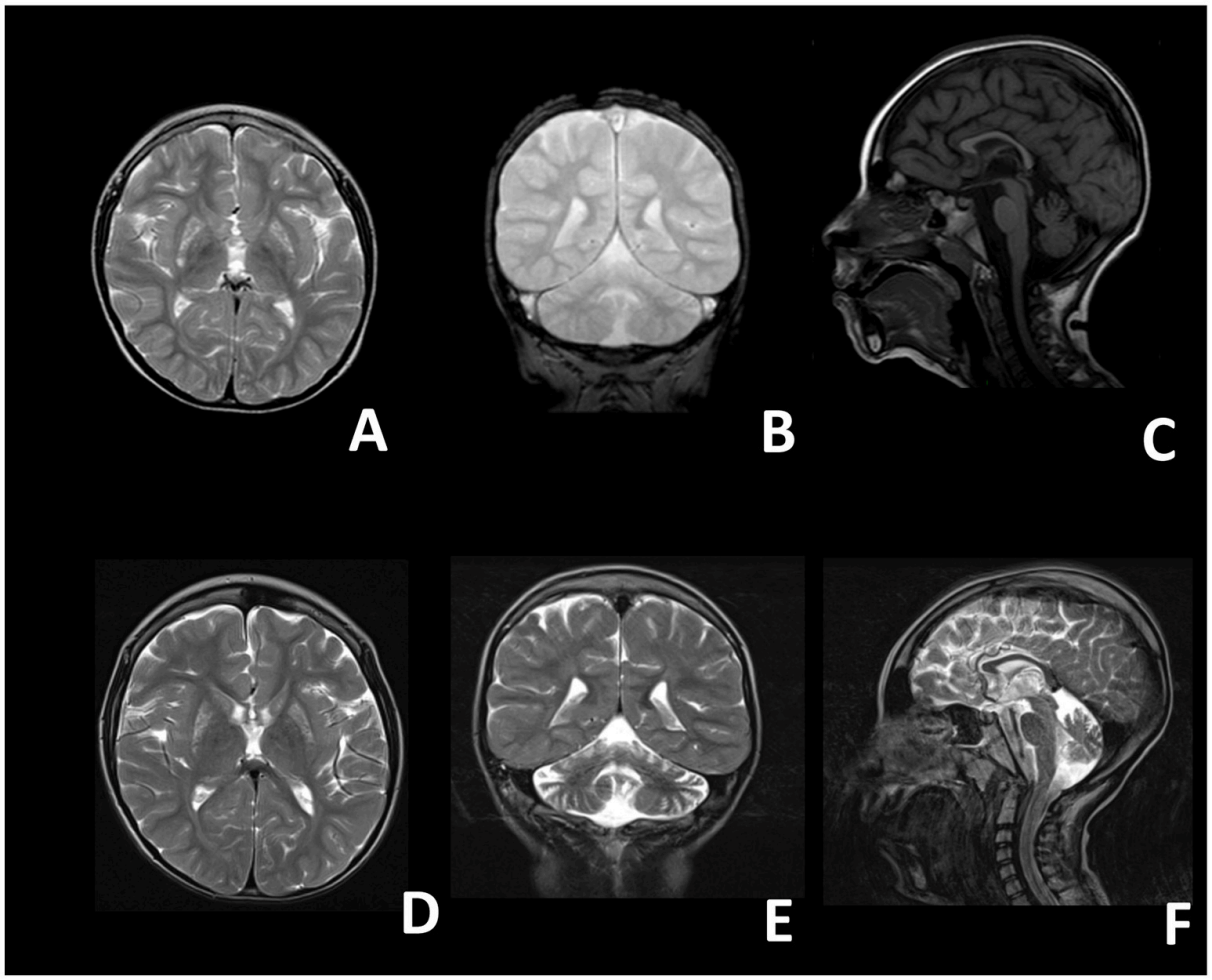
Brain magnetic resonance images of the proband**. (A–C)** Images taken at 4 years; **(D–F)** images taken at 8 years. **(A)** T2 axial, **(B)** T2 coronal, **(C)** T1 sagittal, **(D)** T2 axial, **(E)** T2 coronal, **(F)** T2 sagittal. A-D: bilateral inhomogeneous lesions of the Putamen and of the Caudatum. **(B–E,F)** Progressive cerebellar (vermian and hemispheric) atrophy. **(C–F)** Thin corpus callosum.

At birth, his weight was 2.5 kg (<5th percentile), length was 47 cm (<5th percentile) and head circumference was 33 cm (5th percentile), his postnatal adaptation was normal. Neonatal period was unremarkable. The proband showed a normal psychomotor development until the age of 9 months, when neurological regression was observed. Epileptic seizures emerged at the age of 18 months and were characterized by oro-buccal automatisms, lids myoclonias, hyperventilation with cyanosis and slow anteflexion of the head. At that time, critical EEGs showed rapid activity with rhythmic anteriorly dominant spikes and atypical spike-wave complexes. On evaluation, at 20 months of age, the child presented with microcephaly, failure to thrive, mild dysmorphic features (epicanthus, long philtrum) and exotropia. He appeared hyporeactive and inconstantly responsive when called, with reduced finalistic movements, and showed severe axial hypotonia, with poor head and trunk control, and spasticity with distal dystonic postures of the limbs. In the following years the epileptic phenotype changed into clonic and tonic-clonic seizures associated with oro-buccal automatisms. EEGs were characterized by multifocal spikes, polispikes and atypical spike-wave complexes in a context of disorganized background activity. Mild hepatic steatosis and cholestasis were also detected at 4 years of age, which improved during the course of the years. Last liver ultrasound examination was performed at 10 years of age and didn't show any remarkable alteration. Brain MRIs showed bilateral lesions of putamen, globus pallidus, caudate, and dentate nuclei, compatible with the diagnosis of Leigh Syndrome (Figures 1A–C). On examination at the age of 5 years he showed: severe intellectual disability, microcephaly, spastic-dystonic tetraparesis, severe optic atrophy, and ophthalmoplegia. An extensive metabolic work-up showed lactic acidosis (maximum value: 3.2 mmol/l; reference values 0.5–1.2 mmol/L), and increased lactate in CSF (26.23 mg/dL; reference values 2.25–10 mg/dL) and in the brain (by ^1^H-MRS); very long chain fatty acids (VLCFA), phytanic and pristanic acids, docosahexaenoic acid (DHA), di- and trihydroxycholestanoic acid (DHCA and THCA) were normal. Respiratory chain complex activities in muscle biopsy and fibroblasts, as well as pyruvate dehydrogenase (PDH) activity in fibroblasts, were in the control range. Bioenergetic parameters, such as the maximal respiration rate, index of electron transport chain efficiency (Invernizzi et al., 2012) measured in patient's fibroblasts were similar to controls. Over time his clinical conditions progressively worsened, with severe reduction of the child's reactivity to outer stimuli, a progressive worsening of the spasticity with loss of head and trunk control and a reduction of the spontaneous finalistic movements. Likewise, serial MRIs showed a progressive worsening of brain lesions, with extension of the previously described alterations and severe atrophy of corpus callosum and cerebellum (Figures 1D–F). He suffered from epileptic seizures characterized by focal lip myoclonus, startle reaction and hypertonia of the limbs triggered by the awakening and sudden acoustic stimuli. Asleep EEGs showed multifocal epileptic discharges. Suspecting a mitochondrial disorder, he started antioxidant therapy with riboflavin (100 mg/day), thiamine (100 mg/kg/day), carnitine (100 mg/kg/day).

### Genetic Analysis

**Figure 2:**
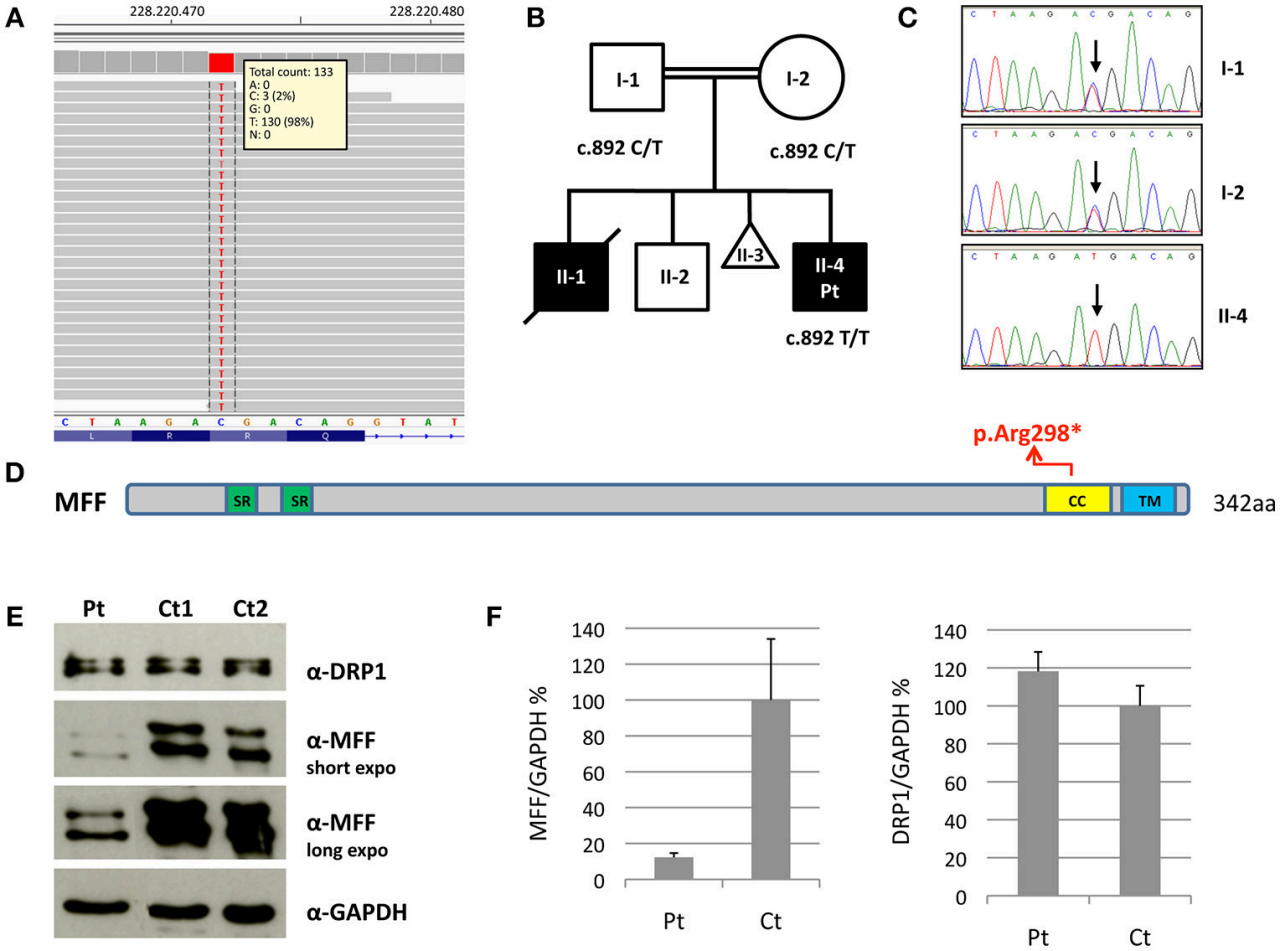
Molecular and protein studies in a patient with *MFF* mutation. **(A)** Snapshot from IGV software of the mutation identified in the proband. **(B,C)** Pedigree of the family and electropherograms of the *MFF* region containing the c.892C>T variant. The black symbols indicate the clinically affected subjects. **(D)** Schematic representation of the MFF protein. Functional domains are in color: Short repeat (SR) in green; coiled-coil (CC) in yellow; transmembrane (TM) in blue. **(E)** Immunoblot analysis of total lysates from control subjects (Ct1 and Ct2) and patient's (Pt) fibroblasts using α-MFF, α-DRP1 and α-GAPDH antibodies. The latter was used as loading control. Two different exposure times are reported for MFF. **(F)** Densitometric analysis of the immunoblots. The levels of MFF and DRP1 proteins were normalized against GAPDH and expressed as percentage relative to the average of all controls. Data are presented as mean ± SD of three experiments.

Molecular analysis for mitochondrial DNA mutations associated with MERRF (m.8344A>G) and NARP (m.8993T>G/T>C) syndromes, and the complete sequencing of *MT-ATP6* and *MT-ATP8* was negative. Because of the clinical presentation suggesting a mitochondrial disorder, targeted resequencing of a panel containing nuclear genes associated with mitochondrial diseases was performed. Given the consanguinity of the parents and the presence of two affected siblings, we hypothesized a recessive trait and focused on rare homozygous variants: only one nucleotide change was identified, c.892C>T, in *MFF* (NM_020194). The variant was validated by Sanger sequencing and found to be heterozygous in both parents. The c.892C>T nucleotide substitution is predicted to cause the synthesis of a truncated protein, p.Arg298^*^ (Figures 2A–D). It was present in only 1 out of >120000 alleles in control population (ExAC database), and already reported in a patient, who carried this nonsense mutation compound heterozygous with a second frameshift change (Koch et al., 2016).

### Protein Studies

We evaluated MFF protein levels in lysates obtained from fibroblasts; we used a polyclonal antibody against MFF that recognizes multiple isoforms of MFF protein due to alternative splicing (band size around 26–29 kDa and 35–38 kDa). Western blot analysis showed the presence of truncated MFF isoforms in the patient, who carries the homozygous variant p.Arg298^*^, and reduction of total amount of MFF compared to controls. Notably, the level of DRP1 was unaffected (Figures 2E,F).

### Morphology Analysis on Patient's Fibroblasts

**Figure 3:**
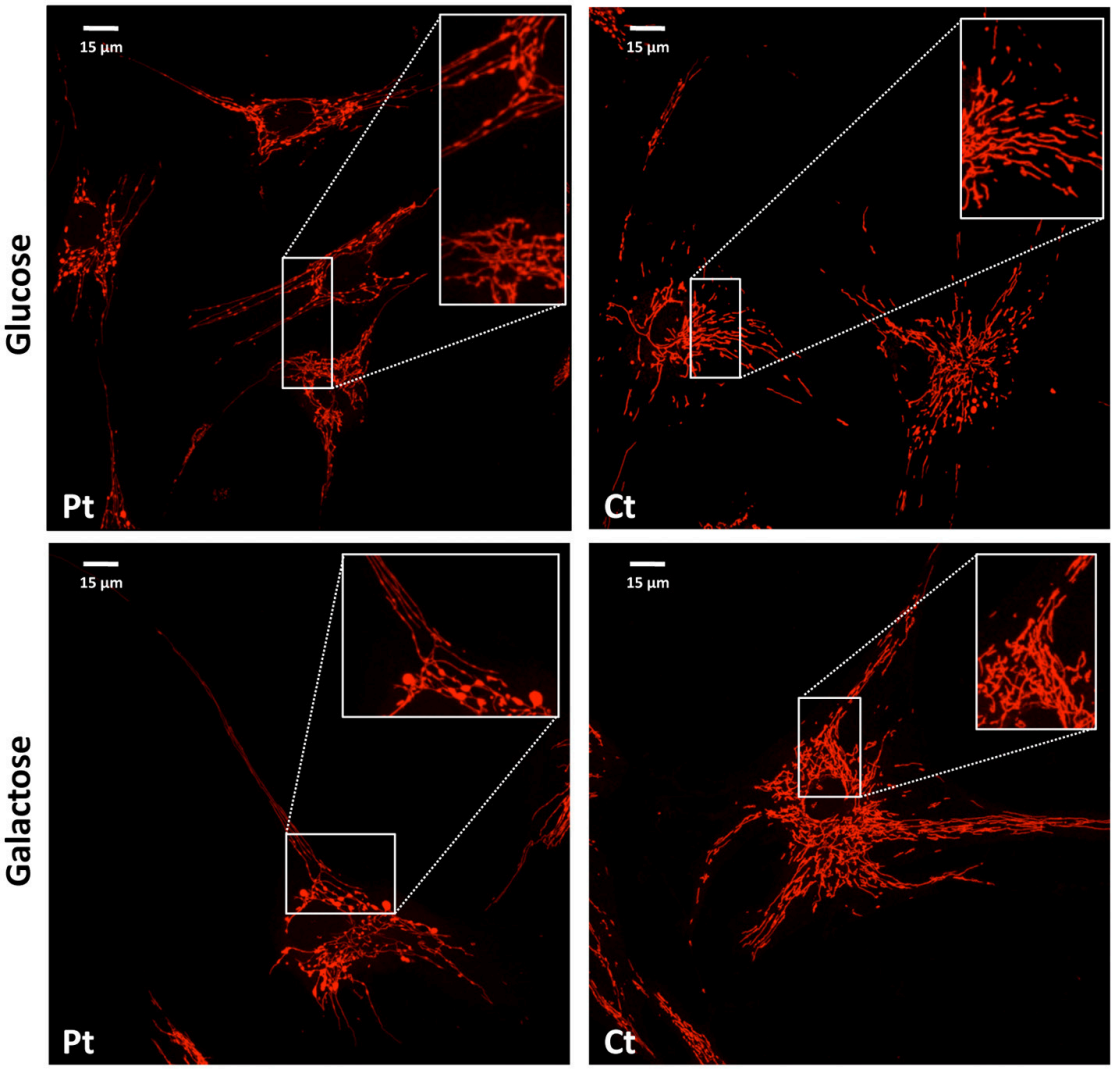
Mitochondrial network analysis in patient fibroblasts. Representative images of the mitochondrial network in fibroblasts from the patient (Pt) and a control subject (Ct), cultured in either glucose **(Upper)** or galactose **(Lower)** medium. In the inset, a digital magnification (3x) shows the peculiar ‘chain-like' structure observed in Pt cells. Scale bar: 15 μm.

We performed microscopy fluorescence studies on fibroblasts and analyzed subcellular organelles by imaging software. First, we stained mitochondria with a specific mitochondrial dye (Mitotracker red CMXRos Invitrogen); we observed a filamentous mitochondrial network in both patient and control fibroblasts, with a similar value of circularity shape factor, indicating an elongated network. Interestingly, at higher magnification, the network of patient cells showed a “chain-like” structure, not observed in controls (Figure 3), but already reported in other mitochondrial fission defects such as those due to mutations in *DNM1L* (Nasca et al., 2016). This finding could indicate that the fission mechanism was initialized but not concluded because of the loss of functional MFF. These morphological alterations were even more evident after cell growth in galactose medium, a condition that forces cells to use mitochondrial respiratory chain to produce ATP rather than glycolysis (Figure 3).

**Figure 4:**
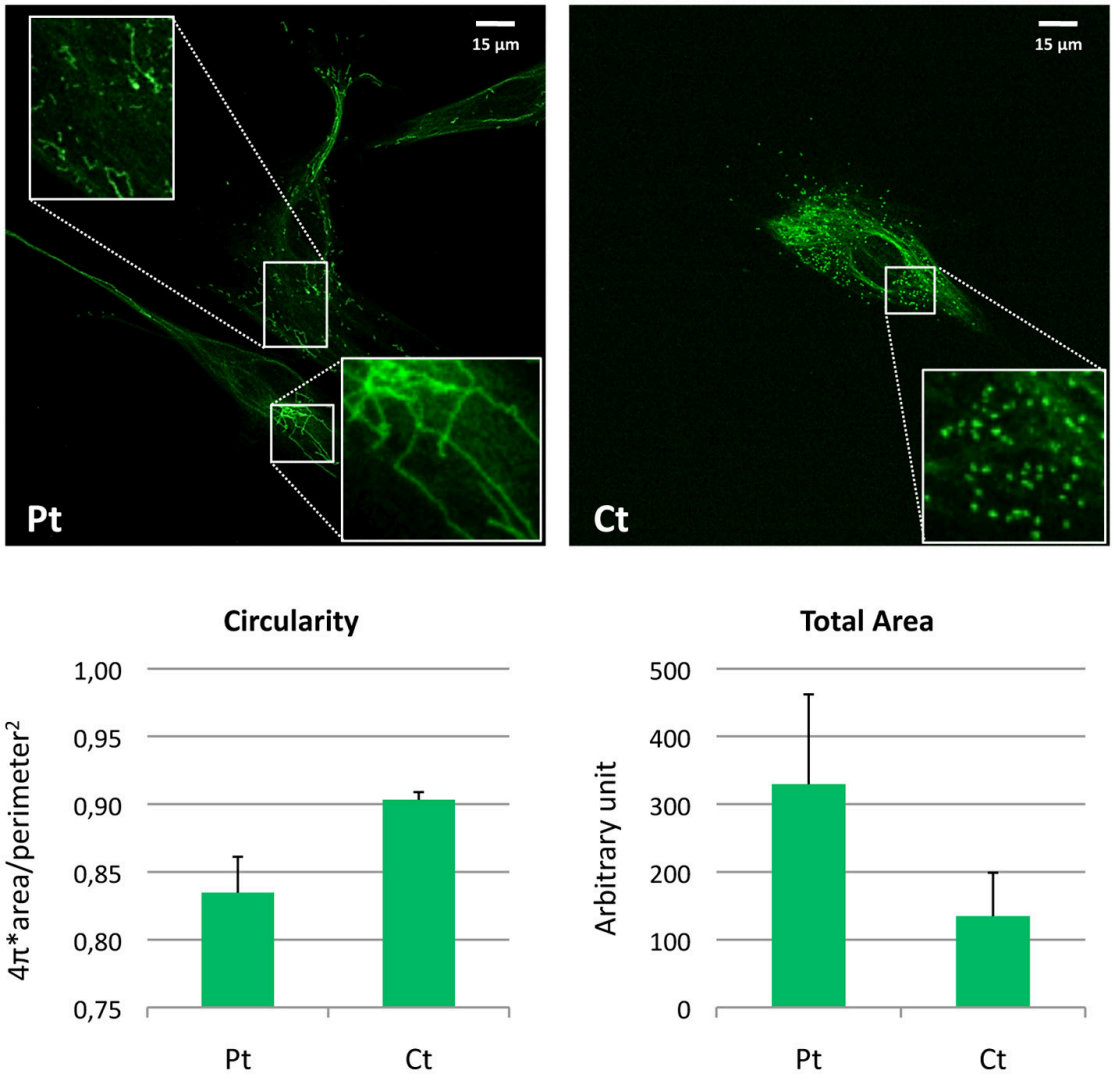
Morphometric analysis of peroxisomal staining in patient fibroblasts. Immunofluorescence staining with the anti-PMP70 antibody in fibroblasts from the patient (Pt) and a control subject (Ct). In the insets, a digital magnification (3x) better shows the altered peroxisomal morphology observed in Pt cells. Scale bar: 15 μm. Analysis of the shape factor (circularity) and mean area of the peroxisomal staining in fibroblasts from Pt and Ct are reported in the graphs. Data are presented as mean ± SD.

For visualization of peroxisomes, we performed immunofluorescence staining using an antibody against PMP70, a peroxisomal membrane protein. In contrast with the normally punctuated staining observed in control fibroblasts, patient's cells revealed very elongated organelles, almost filamentous. Morphometric analysis showed reduced circularity shape factor and increased area in the patient's peroxisomes compared to control (Figure 4).

## Discussion

*MFF* mutations are extremely rare and only five patients from 3 families have been described till now (Table 1). The first cases were two brothers, in whom the genetic cause was identified by autozygome analysis and exome sequencing being part of a cohort of consanguineous cases of mitochondrial disease (Shamseldin et al., 2012). These two patients carried a homozygous nonsense mutation (p.Gln64^*^); marked difference in the mitochondrial and peroxisomal morphology was observed in patient fibroblasts, indicating that *MFF* defect was the cause of the disease.

**Table 1:** Genetic findings and biochemical and phenotypic features of published cases with *MFF* mutations.

**Patient**	P1 (Shamseldin et al., 2012)^*^	**P1 (Koch et al., 2016)**	P2 (Koch et al., 2016)^**^	P3 (Koch et al., 2016)^**^	**Present paper**
Consanguinity	+	–	+	+	+
Origin	Saudi Arabian	Austrian	Turkish	Turkish	Italian
*MFF* variants	c.(190C>T);(190C>T) p.(Gln64^*^);(Gln64^*^)	c.(184dup);(892C>T) p.(Leu62Profs^*^13);(Arg298^*^)	c.(453_454del); (453_454del) p.(Glu153Alafs^*^5); (Glu153Alafs^*^5)	c.(453_454del); (453_454del) p.(Glu153Alafs^*^5); (Glu153Alafs^*^5)	c.(892C>T);(892C>T) p.(Arg298^*^);(Arg298^*^)
**PHENOTYPIC FEATURES**					
Sex	Male	Male	Male	Male	Male
Age at onset	First year	4 months	4 months	11 months	9 months
Microcephaly	+	+	+	+	+
Regression/loss of skills	n.c.	+	+	+	+
Severe developmental delay/intellectual disability	+	+	+	+	+
Spasticity	+	+	+	+	+
Growth retardation	n.c.	–	–	+	+
West syndrome	–	+	+	–	-
Other epileptic and developmental encephalopathies	+	–	–	–	+
External ophthalmoparesis	–	+	+	+	+
Optic athopy	+	+	–(9 months)	+	+
Peripheral neuropathy	n.c.	+	n.d.	+	-
Movement disorder	-	-	-	-	Upper limb dystonia
Brain MRI lesions	Pallidum	Putamen, pallidum, caudate, mesencephalon, dentate nucleus optic radiation, cerebellar atrophy	Pallidum, mesencephalon, thalamus	Putamen, pallidum, caudate, thalamus, mesencephalon, cerebellar atrophy	Putamen, caudate, mesencephalon, cerebellar atrophy
Increased brain la ctate (liquor/MRS)	n.c.	–	n.d.	n.d.	+
**LABORATORY FINDINGS**					
Increased plasma lactate	–	+	+	+	+
MRC in muscle	n.d.	Normal	n.d.	Normal	Slight increased SDH
MRC fibroblasts	Normal	n.d.	n.d.	n.d.	Normal
VLCFA increased	–	–	–	n.d.	–
Plasmalogens abnormal	n.d.	–	n.d.	n.d.	–
Pristanic acid abnormal	n.d.	–	n.d.	n.d.	–
Phytanic acid abnormal	n.d.	–	n.d.	n.d.	–
Bile acid metabolites abnormal	n.d.	–	n.d.	n.d.	–
Bone abnormalities	n.d.	–	n.d.	n.d.	Osteoporosis
Kidney cysts	n.d.	–	n.d.	n.d.	–

*+, present; –, absent; CSF, cerebrospinal fluid; GMFCS, Gross Motor Function Classification System; MFF, mitochondrial fission factor; MRC, mitochondrial respiratory chain enzymes; MRS, magnetic resonance spectroscopy; n.c., not commented; n.d., not determined; VLCFA, very long chain fatty acids*.

* *A younger brother was reported as similarly delayed but without any further description*.

** *These cases are two siblings*.

A singleton case and two additional siblings with *MFF* mutations were later described (Koch et al., 2016). Main features of these patients are early onset epileptic encephalopathy, acquired microcephaly, optic atrophy, external ophthalmoparesis, neuroradiological findings evocative of Leigh syndrome, all together suggesting mitochondrial dysfunction without impairment of the mitochondrial respiratory chain (more details in Table 1).

Our patient is the first reported case with such a long neurological follow-up. His clinical phenotype is quite similar to previously reported cases, but the progression of symptoms was slower; at 11 years of age only a mild dysphagia was detected. Although his neurological phenotype was dominated by spasticity, dystonic postures of the upper limb extremities were present. Interestingly, an anti-oxidant therapy was started at the age of 3, with a slight improvement of the general conditions of the child. No detail about the use of anti-oxidant therapy in this disorder was available in previous reports. Remarkably, hepatic steatosis gradually remitted over the years and, at the age of 11, the patient did not present any notable hepatic functionality alteration.

Mutations of genes involved in mitodynamics (e.g., *OPA1, DNM1L*…) may lead to overlapping clinical presentations often including optic atrophy as isolated symptom or part of severe multisystem disorders. This evidence has been explained by a peculiar vulnerability of retinal ganglion cells to mitochondrial dysfunction. Given the accessibility of the eye as a part of the central nervous system, ophthalmologic examination has been suggested for correct diagnosis of these complex clinical entities (Bagli et al., 2017).

Our patient showed clinical features suggesting both mitochondrial and peroxisomal damage but biochemical analyses were less informative. Lactate was found elevated in CSF and by brain spectroscopy, and moderately high on plasma, but neither respiratory chain defects nor biomarkers of peroxisomal dysfunction were observed. Similar results were obtained in the other *MFF* patients, with plasma lactate only occasionally increased and other biochemical values in the control range.

The absence of overt mitochondrial and peroxisomal biochemical impairment is in line with what is reported in other disorders affecting the dynamics of intracellular organelles (e.g. *DNM1L* mutations). Mitochondrial diseases typically affect neurons because they are among the most energy-consuming cell types; however, in addition to bioenergetics defects, there are evidences indicating a key role of impaired mitodynamics in neurodegenerative diseases, in particular mitochondrial transport along axons. A block in mitochondrial fission (e.g., because of mutations in *MFF* or *DNM1L*), causing the formation of larger organelles, may have deleterious effects on the mobility of mitochondria, on synaptic homeostasis, on mitophagy (Sheng and Cai, 2012; Itoh et al., 2013; Burté et al., 2015; Pellegrino and Haynes, 2015).

Accordingly, the analysis performed on fibroblasts from our patient (present paper) and other subjects with *MFF* mutations (Shamseldin et al., 2012; Koch et al., 2016) clearly showed that *MFF* alterations caused derangement of the morphology of mitochondria and peroxisomes; this was particularly evident in the latters, with an extremely filamentous appearance which was more striking than the elongated peroxisomes observed in *DNM1L* patients (Nasca et al., 2016). These findings confirmed a crucial role for MFF in fission of these organelles.

## Author Contributions

FN, AC, MS, and VL obtained clinical, biochemical and radiological data. BG and DG conceived and designed the experiments. AN and AL performed the experiments. AN, FN, DG, and VL wrote the paper. All authors read and approved the manuscript.

### Conflict of Interest Statement

The authors declare that the research was conducted in the absence of any commercial or financial relationships that could be construed as a potential conflict of interest. The handling editor declared a past collaboration with the authors AN and DG.
